# *Rhodococcus daqingensis* sp. nov., isolated from petroleum-contaminated soil

**DOI:** 10.1007/s10482-018-1201-y

**Published:** 2018-11-22

**Authors:** Lili Wang, Liguo Zhang, Xiaofei Zhang, Shuquan Zhang, Lei Yang, Hongmei Yuan, Jing Chen, Chunbo Liang, Wengong Huang, Jianxin Liu, Yuanling Zhao, Qian Yang

**Affiliations:** 1 School of Life Science and Technology, Harbin Institute of Technology, Harbin, China; 2 Heilongjiang Academy of Agricultural Sciences, Harbin, China; 3 College of Mathematics and Information Science of Guangxi University, Nanning, China; 4 Department of Mathematics, Harbin Institute of Technology, Harbin, China; 5 Foundation Department, Dalian University of Finance and Economics, Dalian, China

**Keywords:** *Rhodococcus daqingensis* sp.nov., Petroleum-contaminated soil, Polyphasic taxonomy, 16S rRNA gene, DNA–DNA hybridization

## Abstract

A novel Gram-stain positive, aerobic, non-motile bacterial strain, designated Z1^T^, was isolated from a sample of petroleum-contaminated soil collected in Daqing, Heilongjiang province, China and characterised with a series of taxonomic approaches. The morphological and chemotaxonomic properties of the isolate were typical of those of members of the genus *Rhodococcus*. Phylogenetic analyses based on 16S rRNA gene sequences showed that strain Z1^T^ belongs to the genus *Rhodococcus* and clustered with *Rhodococcus maanshanensis* DSM 44675^T^ (99.2%, sequence similarity) and *Rhodococcus tukisamuensis* JCM 11308^T^ (97.9%), respectively. However, the DNA–DNA hybridizations between strain Z1^T^ and *R. maanshanensis* DSM 44675^T^ and *R. tukisamuensis* JCM 11308^T^ were both less than 70%. The optimal growth temperature and pH for strain Z1^T^ were found to be at 28 °C and at pH 7.0. The peptidoglycan was found to contain *meso*-diaminopimelic acid; arabinose, galactose and glucose were detected as diagnostic sugars. The main polar lipids were identified as diphosphatidylglycerol, phosphatidylethanolamine, phosphatidylinositol, phosphatidylinositol mannoside and an unidentified lipid; MK-8(H_2_) was found as the major menaquinone. The major fatty acids were identified as C_16:0_, 10-methyl C_18:0_ and C_18:1_*ω*9*c*. Mycolic acids were found to be present. The G + C content of the genomic DNA was determined to be 66.7 mol%. Based on a comparative analysis of phenotypic and genotypic characteristics, in combination with DNA–DNA hybridization results, strain Z1^T^ can be distinguished from the type strains of its two close neighbours as a novel species of the genus *Rhodococcus*, for which the name *Rhodococcus daqingensis* sp. nov. is proposed. The type strain is Z1^T^ (= CGMCC 1.13630^T^ = DSM 107227^T^).

**Electronic supplementary material:**

The online version of this article (10.1007/s10482-018-1201-y) contains supplementary material, which is available to authorized users.

## Introduction

The genus *Rhodococcus* of the family *Nocardiaceae* of the order *Corynebacteriales* (Jones and Goodfellow 2012), belonging to the phylum *Actinobacteria*, was first described by Zopf (1891), then refined by Goodfellow and Alderson (1977) and emended subsequently by Goodfellow et al. (1998). With the results of recent taxogenomic studies, the genus *Rhodococcus* has been shown to be a highly polyphyletic taxon and includes at least seven distinct subgroups (Sangal et al. 2016). The number of species classified within the genus *Rhodococcus* has significantly increased in the last decade, and at the time of writing, more than 50 species with validly published names have been described (http://www.bacterio.net/rhodococcus.html). The growth cycle of members of the genus show a succession of more or less complex rod-coccus morphological stages. Members of the genus *Rhodococcus* are characterised by the presence of MK-8(H_2_) and/or MK-8(H_4_) as the predominant menaquinones; straight-chain saturated, monounsaturated and branched-chain fatty acids as the major fatty acids; phosphatidylethanolamine, diphosphatidylglycerol, phosphatidylinositol and phosphatidylinositol mannosides as major components of the polar lipids. Mycolic acids are present. Whole cell hydrolysates contain *meso*-2, 6-diaminopimelic acid, as the diagnostic diamino acid (Jones and Goodfellow 2012). The DNA G + C content of members of the genus *Rhodococcus* range from 67.0 to 72.0 mol%. They have been isolated from different niches such as soil, water, marine sediment, insect, plants and other sources.

Previous studies have shown that some species of the genus can digest a mixture of hydrocarbons, gasoline and diesel oil (Auffret et al. 2009, 2015???), or hexane and different hydrocarbons, and petroleum hydrocarbons (Lee et al. 2010). During a study of the diversity of microorganisms of a petroleum-contaminated soil, strain Z1^T^ was isolated. In this study, we performed a polyphasic taxonomic study on this strain and conclude that it represents a novel species of the genus *Rhodococcus*, for which the name *Rhodococcus daqingensis* sp. nov. is proposed.

## Materials and methods

### Isolation and maintenance of microorganisms

Strain Z1^T^ was isolated from a sample of petroleum-contaminated soil collected in Daqing City, Heilongjiang province, China (46° 68′ N, 125° 03′ E). The strain was isolated using the standard dilution plate method and grown on humic acid-vitamin agar (HV) (Hayakawa and Nonomura 1987) supplemented with cycloheximide (50 mg l^−1^) and nalidixic acid (20 mg l^−1^). After 21 days of aerobic incubation at 28 °C, selected colonies were transferred and purified on International *Streptomyces* Project (ISP) 2 medium (Shirling and Gottlieb 1966) and maintained in glycerol suspensions (20%, v/v) at − 80 °C.

The type strains of *Rhodococcus maanshanensis* DSM 44675^T^ and *Rhodococcus tukisamuensis* JCM 11308^T^ were purchased from the German Collection of Microorganisms and Cell Cultures (DSMZ, Germany) and Japan Collection of Microorganisms (JCM), respectively. All strains were maintained routinely on ISP 2 medium (28 °C, 1 week). Biomass of strain Z1^T^ and the reference type strains for chemotaxonomic and molecular investigations were harvested from cultures grown on ISP 2 medium (28 °C, 1 week).

### Phenotypic characteristics

Morphological characteristics were observed by light (Nikon ECLIPSE E200) and scanning electron microscopy (JSM-6330F, JEOL) using cultures grown on ISP 2 agar at 28 °C for 1–7 days. Gram-staining was determined by the KOH test (Gregersen 1978). Cell motility was tested by the development of turbidity throughout a tube containing semisolid medium (Leifson 1960). Cultural characteristics were determined on ISP media 2–5 (Shirling and Gottlieb 1966), Czapek’s agar (Waksman 1967), nutrient agar (Waksman 1967) and tryptic soy agar (TSA; Difco). The colony colour was determined using the ISCC-NBS colour chart (Kelly 1964). Growth tests were determined on plates of ISP 2 medium for temperatures from 10 to 60 °C at 5 °C increments and for pH values from 4.0 to 12.0 (in increments of 1.0 pH unit) on ISP2 medium by using the buffer system described by Xie et al. (2012). NaCl tolerance was determined in ISP 2 medium supplemented with 0–10% NaCl (w/v) at 28 °C for 14 days on a rotary shaker. Production of catalase and urease, hydrolysis of starch and Tweens 20, 40, 60 and 80 were tested as described by Smibert and Krieg (1994). The utilisation of sole carbon and nitrogen sources, decomposition of cellulose and urea, reduction of nitrate, peptonisation of milk, liquefaction of gelatin and production of H_2_S were examined as described previously (Gordon et al. 1974; Williams et al. 1983; Yokota et al. 1993). The physiological properties of strain Z1^T^ and the reference type strains were tested using the methods described above.

### Chemotaxonomic characterisation

Biomass for chemical studies was prepared by growing strain Z1^T^ and reference strains in tryptic soy broth shake flasks at 28 °C for 7 days and then cells were freeze-dried. The diaminopimelic acid isomers of the cell wall was determined according to Staneck & Roberts (1974) and the sugars of the whole cell hydrolysate were analysed as described by Tang et al. (2009). Mycolic acids of strain Z1^T^ and the reference type strains were extracted and analysed according to the method described by Minnikin et al. (1980). The polar lipids were examined by two-dimensional TLC and identified with the method described by Minnikin et al. (1984). Menaquinones were extracted from freeze-dried biomass and purified according to Collins (1985). Extracts were analysed using a HPLC–UV method (Wu et al. 1989). Cellular fatty acids were extracted, methylated and analysed following the instructions of the Microbial Identification System (MIDI) (Sherlock Version 6.1; MIDI database: TSBA6) (Sasser 1990). Biomass for fatty acid analysis was obtained from strains grown on TSA at 28 °C for 7 days.

### Molecular analysis

Genomic DNA isolation and PCR amplification of the 16S rRNA gene was performed as described by Li et al. (2007). The almost-complete 16S rRNA gene sequence (1507 bp) of strain Z1^T^ was submitted to the EzTaxon server (Kim et al. 2012) and aligned with the 16S rRNA gene sequences of other *Rhodococcus* species using CLUSTAL X version 2.1 (Larkin et al. 2007). Phylogenetic trees based on the aligned sequences were inferred using the neighbour-joining (Saitou and Nei 1987), maximum likelihood (Felsenstein 1981) and maximum parsimony (Fitch 1971) trees generated with MEGA 7.0 (Kumar et al. 2016). The stability of the topology of the phylogenetic trees was assessed using the bootstrap method with 1000 repetitions (Felsenstein 1985). A distance matrix was generated using Kimura’s two-parameter model (Kimura 1980). All positions containing gaps and missing data were eliminated from the dataset (complete deletion option).

The G + C contents of the genomic DNA were determined by the thermal denaturation (*Tm*) method (Mandel and Marmur 1968). DNA–DNA hybridization tests were carried out as described by De Ley et al. (1970) with consideration of the modifications described by Huss et al. (1983), using a model Cary 100 Bio UV/VIS-spectrophotometer equipped with a Peltier-thermostatted 6 × 6 multicell changer and a temperature controller with in situ temperature probe (Varian). The experiments were performed with three replications and the DNA–DNA relatedness values were expressed as mean of the three values.

## Results and discussion

Strain Z1^T^ has chemotaxonomic, morphological and phenotypic properties typical of the members of the genus *Rhodococcus*. Strain Z1^T^ was observed to be Gram-stain positive, aerobic, non-acid-fast and non-motile. Morphological observation of a 7-day-old culture of strain Z1^T^ grown on ISP 2 agar revealed that it forms circular, opaque, convex and grayish pink colonies with irregular edges. Scanning electron micrographs of strain Z1^T^ at different time intervals showed that the cells follow a distinct rod-coccus cycle during growth stages, which is consistent with its assignment to the genus *Rhodococcus* (Supplementary Fig. S1). Good growth was observed on Czapek’s agar, nutrient agar, TSA, ISP 2 and ISP 3 media; poor growth was observed on ISP 4 and ISP 5 media. Strain Z1^T^ was observed to grow well between pH 6.0–9.0, with an optimum pH of 7.0. The range of temperature for growth was determined to be 15–40 °C, with the optimum growth temperature at 28 °C. Strain Z1^T^ was observed to grow in the presence of 0–4% NaCl (w/v). The physiological and biochemical characteristics of strain Z1^T^ are shown in the species description.

Strain Z1^T^ was found to contain *meso*-diaminopimelic acid as the cell wall diamino acid. The whole cell hydrolysate was found to contain arabinose, galactose and glucose. MK-8(H_2_) was the only menaquinone detected. The polar lipids profile was found to consist of diphosphatidylglycerol, phosphatidylethanolamine, phosphatidylinositol, phosphatidylinositol mannoside and an unidentified lipid (Supplementary Fig. S2). The predominant fatty acids identified in strain Z1^T^ (> 10%) were C_16:0_ (33.6%), 10-methyl C_18:0_ (20.7%) and C_18:1_ω9c (13.2%). Detailed fatty acid compositions are shown in Table 1. Mycolic acids were found to be present (Rf value = 0.45; Supplementary Fig. S3). These chemotaxonomic data are consistent with the assignment of strain Z1^T^ to the genus *Rhodococcus* (Jones and Goodfellow 2012).

**Table 1:** Fatty acid profiles of strain Z1^T^ and the reference type strains of the genus *Rhodococcus*

Fatty acids	1	2	3
Saturated			
C_14:0_	3.5	3.6	4.7
C_15:0_	2.7	6.3	3.1
C_16:0_	33.6	30.8	35.1
C_17:0_	2.9	3.9	1.2
C_18:0_	0.9	1.6	1.1
Bracnched			
iso-C_14:0_	0.6	0.7	–
iso-C_16:0_	0.7	–	1.0
iso-C_17:0_	0.9	0.7	0.5
Unsaturated			
C_17:1_*ω*9*c*	5.5	6.4	11.8
C_18:1_*ω*9*c*	13.2	16.3	15.9
10-Methyl			
C_18:0_ 10-methyl	20.7	15.9	8.4

Strains: 1, Z1^T^; 2, *R. maanshanensis* DSM 44675^T^; 3, *R. tukisamuensis* JCM 11308^T^

All data were obtained from this study. Biomass was harvested from TSA broth at 28 °C when the growth was half of the maximum value. Values are percentage of total fatty acids. –, not detected. Fatty acids that represent < 0.5% in all strains were omitted

**Fig. 1:**
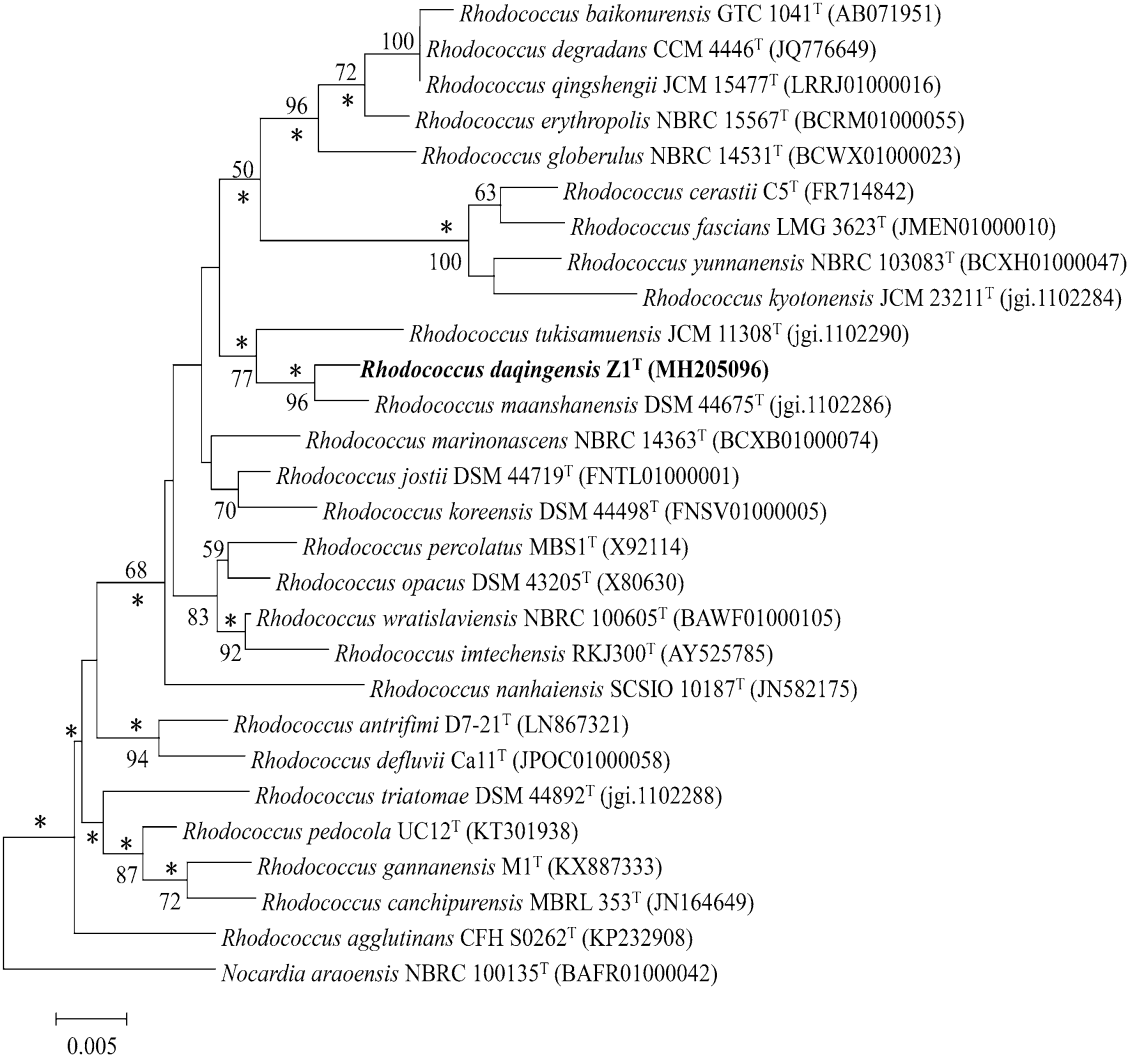
Neighbour-joining tree based on 16S rRNA gene sequences (1507 bp), showing the relationship of strain Z1^T^ and type strains of species of the genus *Rhodococcus*. Bootstrap values > 50% (based on 1000 replications) are shown at branch points. *Nocardia araoensis* NBRC 100135^T^ was used as an outgroup. Asterisks indicate that the corresponding nodes (groupings) are also recovered using the maximum likelihood and maximum-parsimony methods. Bar, 0.005 substitutions per nucleotide position

The almost-complete 16S rRNA gene sequence of strain Z1^T^ was determined (1507 bp) and deposited in the GenBank/EMBL/DDBJ databases as MH205096. Identification of the close phylogenetic neighbours confirmed the phylogenetic affiliation to the genus *Rhodococcus*. The 16S rRNA gene sequence of strain Z1^T^ showed a close relationship with those of *R. maanshanensis* DSM 44675^T^ (99.2%, sequence similarity) and *R. tukisamuensis* JCM 11308^T^ (97.9%). The neighbour-joining phylogenetic tree based on 16S rRNA gene sequences showed that strain Z1^T^ forms a distinct phyletic cluster with its close neighbours *R. maanshanensis* DSM 44675^T^ and *R. tukisamuensis* JCM 11308^T^ (Fig. 1). This apparently distinct lineage was also supported by the maximum-likelihood and maximum-parsimony trees (Supplementary Fig. S4 a,b). *R. maanshanensis* and *R. tukisamuensis* have previously been shown to cluster together in phylogenomic analyses (Sangal et al. 2016). The genomic DNA G + C content of strain Z1^T^ was determined to be 66.7 mol%, which is within the range for members of the genus *Rhodococcus* (Jones and Goodfellow 2012). Based on the phylogenetic trees and EzTaxon-e server results, DNA–DNA hybridization was carried out between strain Z1^T^ and *R. maanshanensis* DSM 44675^T^ and *R. tukisamuensis* JCM 11308^T^, and the level of DNA–DNA relatedness between these strains were 51.8 ± 0.3% and 31.6 ± 0.7%, respectively. These values are below the threshold value of 70% recommended by Wayne et al. (1987) for assigning bacteria to the same genomic species.

The phylogenetic analyses, morphological and chemotaxonomic characteristics support the classification of strain Z1^T^ as a member of the genus *Rhodococcus*. However, the strain can be differentiated from the closely related strain *R. maanshanensis* DSM 44675^T^ and *R. tukisamuensis* JCM 11308^T^ by several biochemical characteristics, besides the low DNA–DNA relatedness values. Strain Z1^T^ cannot degrade Tweens 20 and 80, which the reference strains can. Strain Z1^T^ is positive for oxidase activity, whereas the two related type strains are not. Moreover, differences were observed in the utilisation of sole carbon and nitrogen sources between strains Z1^T^ and two related type strains (Table 2).

**Table 2:** Differential characteristics of strain Z1^T^ and type strain of the closely related species of the genus *Rhodococcus*

Characteristics	1	2	3
Ranges for growth			
Temperature (°C)	15–40	20–40	15–45
pH	6.0–9.0	6.0–8.0	5.0–9.0
NaCl	0–4%	0–5%	0–3%
Hydrolysis of			
Tween 20	–	+	+
Tween 60	+	+	–
Tween 80	–	+	+
Oxidase	+	–	–
Utilisation as sole carbon source			
d-fructose	+	+	–
Maltose	–	+	+
d-ribose	+	+	–
l-sorbose	–	+	–
d-xylose	+	–	–
Utilization as sole nitrogen source			
l-alanine	+	+	–
l-asparagine	–	–	+
l-serine	+	–	–
Characteristic sugars	Arabinose, galactose and glucose	Arabinose and galactose	Arabinose and galactose
Polar lipids	DPG, PE, PI, PIM, L	DPG, PE, PIM	DPG, PE, PIM
Major fatty acids (> 10%)	C_16:0_, 10-methyl C_18:0_, C_18:1_*ω*9*c*	C_16:0_, C_18:1_*ω*9*c*, 10-methyl C_18:0_	C_16:0_, C_18:1_*ω*9*c*, C_17:1_*ω*9*c*
DNA G + C content (mol%)	66.7	66.2	66.8

Strains: 1, Z1^T^; 2, *R. maanshanensis* DSM 44675^T^; 3, *R. tukisamuensis* JCM 11308^T^. All physiological data are from this study. +, positive reaction; −, negative reaction

Based on the above characteristics, strain Z1^T^ is considered to represent a novel species of genus *Rhodococcus*, for which the name *Rhodococcus daqingensis* sp. nov. is proposed. The Digital Protologue database (Rosselló-Móra et al. 2017) Taxon Number for strain Z1^T^ is TA00704.

## Description of *Rhodococcus daqingensis* sp. nov.

*Rhodococcus daqingensis* (da.qing.en’sis. N.L. masc. adj. *daqingensis*, pertaining to Daqing City, China, where the type strain was isolated).

Gram-stain positive, aerobic, non-acid-fast and non-motile. Cells show a rod-coccus cycle during growth. Colonies are circular, opaque, convex and grayish pink with irregular edges. Growth occurs at 15–40 °C and pH 6.0–9.0, with optimum growth at 28 °C and pH 7.0. Growth occurs in the presence of up to 4% NaCl. Positive for catalase and oxidase tests and for hydrolysis of Tween 60. Negative for hydrolysis of starch, Tween 20, Tween 40, Tween 80, reduction of nitrate, decomposition of cellulose and urease, liquefaction of gelatin, production of H_2_S and peptonisation of milk. d-fructose, d-galactose, d-glucose, d-mannose, raffinose, d-ribose, d-sorbitol and d-xylose are utilised as sole carbon sources but l-arabinose, *myo*-inositol, lactose, maltose, mannitol, rhamnose and l-sucrose are not. l-alanine, l-arginine, l-glutamic acid, l-glutamine, glycine, l-serine and l-valine are utilised as sole nitrogen sources but l-asparagine, l-histidine, and threonine are not. The diagnostic amino acid of the cell wall is *meso*-diaminopimelic acid. Whole cell hydrolysates contain arabinose, galactose and glucose. The predominant menaquinone is MK-8(H_2_). The main polar lipids are diphosphatidylglycerol, phosphatidylethanolamine, phosphatidylinositol, phosphatidylinositol mannoside and an unidentified lipid. Major fatty acids (> 10.0%) are C_16:0_, 10-methyl C_18:0_ and C_18:1_*ω*9*c*. The DNA G + C content of the type strain is 66.7 mol%.

The type strain is Z1^T^ (= CGMCC 1.13630^T^ = DSM 107227^T^), which was isolated from a sample of petroleum-contaminated soil collected in Daqing City, Heilongjiang province, China. The GenBank/EMBL/DDBJ accession number for the 16S rRNA gene sequence of strain Z1^T^ is MH205096.

## Electronic supplementary material

Below is the link to the electronic supplementary material.

Supplementary material 1 (DOCX 2659 kb)
