# Supplementary material for: Rhodococcus daqingensis sp. nov., isolated from petroleum-contaminated soil
Source: Antonie Van Leeuwenhoek. 2018 Nov 22;112(5):695–702. doi: 10.1007/s10482-018-1201-y (PMC6456462; doi:10.1007/s10482-018-1201-y)
Supplement: Supplementary file 1 — Supplementary material 1 (DOCX 2659 kb) [file 10482_2018_1201_MOESM1_ESM.docx]

**Fig. S1.** Scanning electron micrographs of strain Z1^T^ grown on ISP 2 agar at 28 ^o^C for different growth phases. a, 24 h; b, 48 h; c, 72 h. Bar, 1μm.


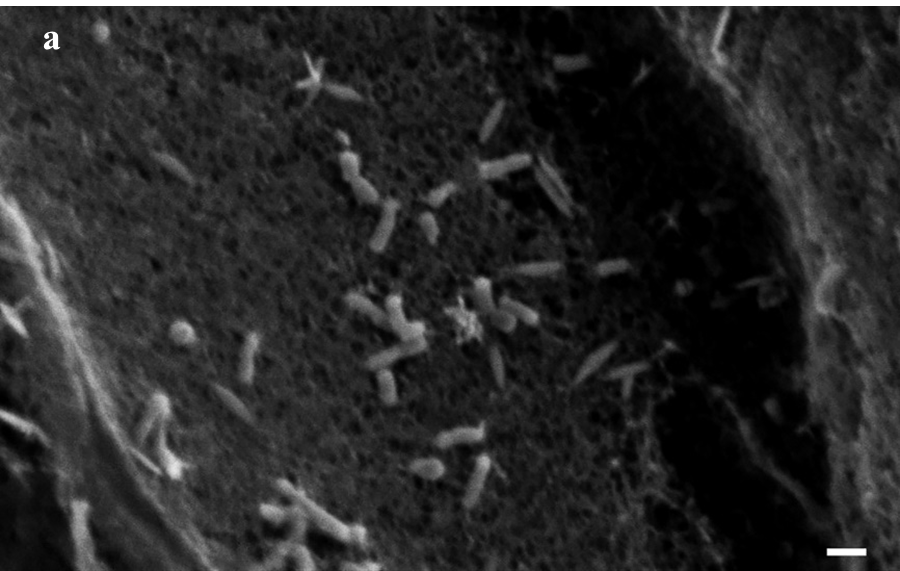


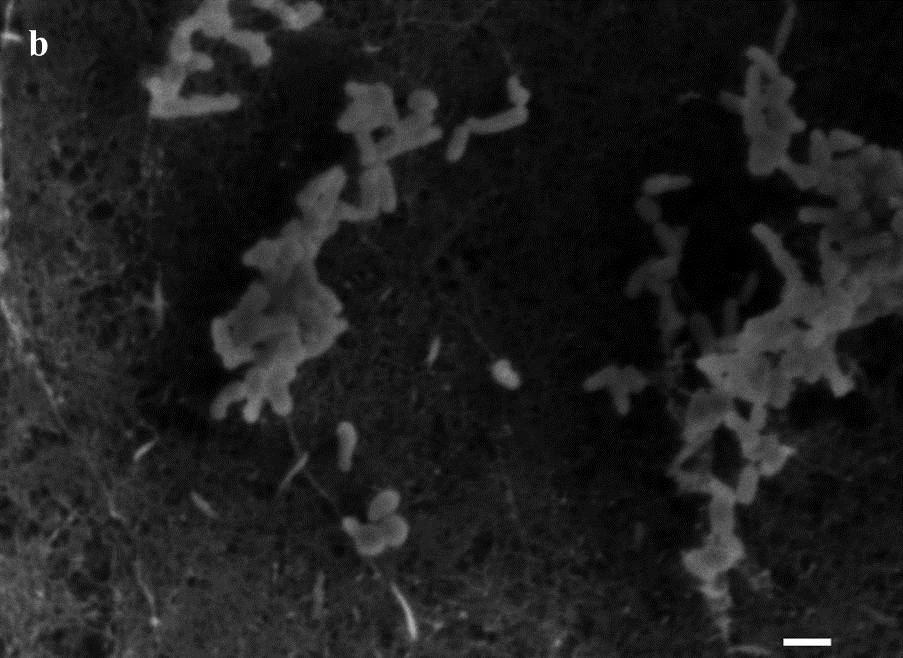


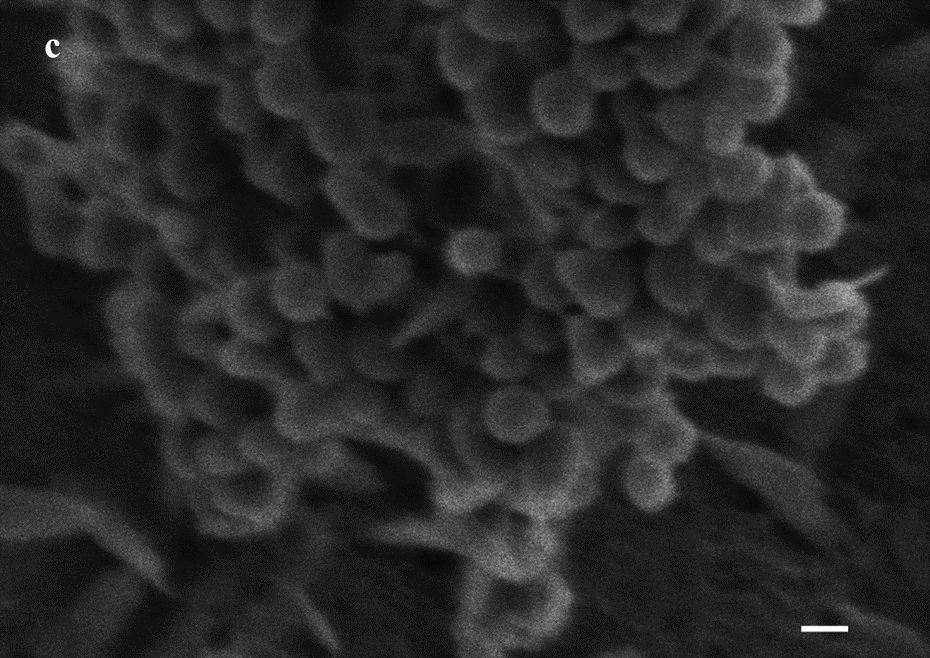


**Fig. S1**

**Fig. S2.** The phospholipids of strain Z1^T^ after two-dimensional TLC. Diphosphatidylglycerol (DPG), phosphatidylethanolamine (PE), phosphatidylinositol (PI), phosphatidylinositol mannoside (PIM) and Unidentified lipids (L). Using molybdophosphoric acid reagent.

**
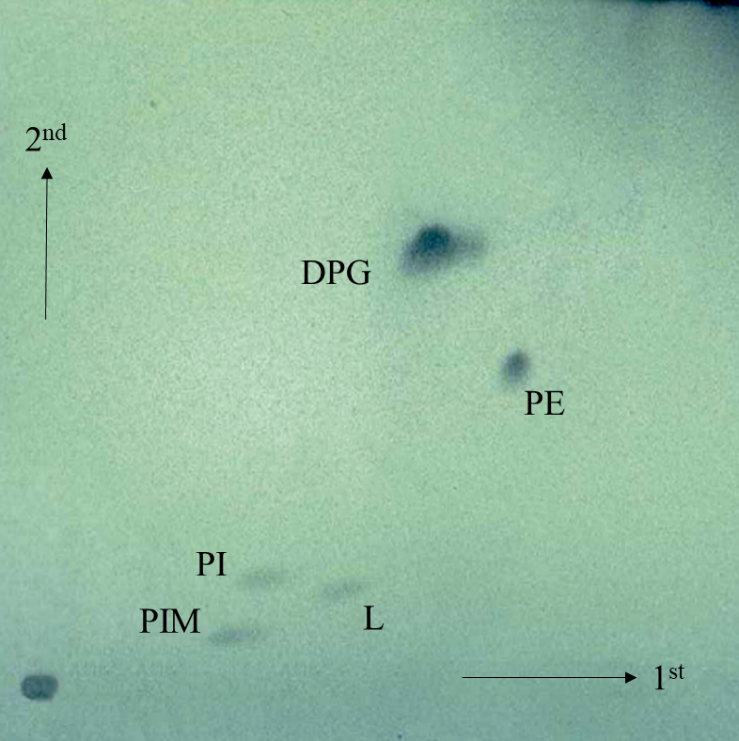
**

**Fig. S2**

**Fig. S3.** The thin-layer chromatogram of mycolic acid of strain Z1^T^ and the reference strains stained with 10 % ethanolic molybdophosphoric acid.

Note: M, mycolic acid

**
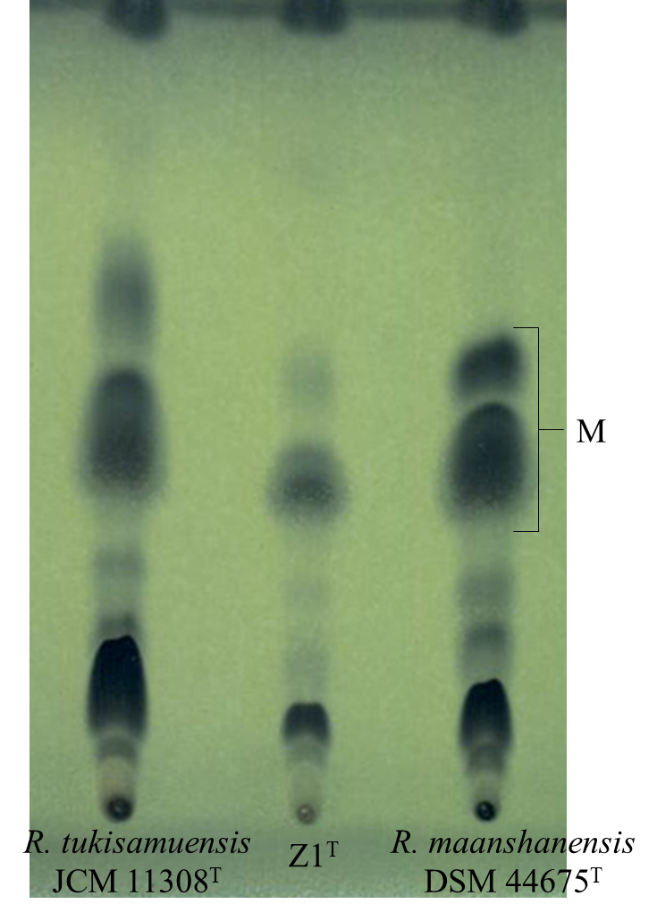
**

**Fig. S3**

**Fig. S4.** Maximum likelihood tree and maximum-parsimony tree based on 16S rRNA gene sequences (1507 bp), showing the relationship between strain Z1^T^ and type strains of species of the genus *Rhodococcus*. Bootstrap values >50% (based on 1000 replications) are shown at branch points. *Nocardia araoensis* NBRC 100135^T^ was used as an outgroup. Bar, 0.005 substitutions per nucleotide position. a, Maximum likelihood tree; b, Maximum-parsimony tree.

**
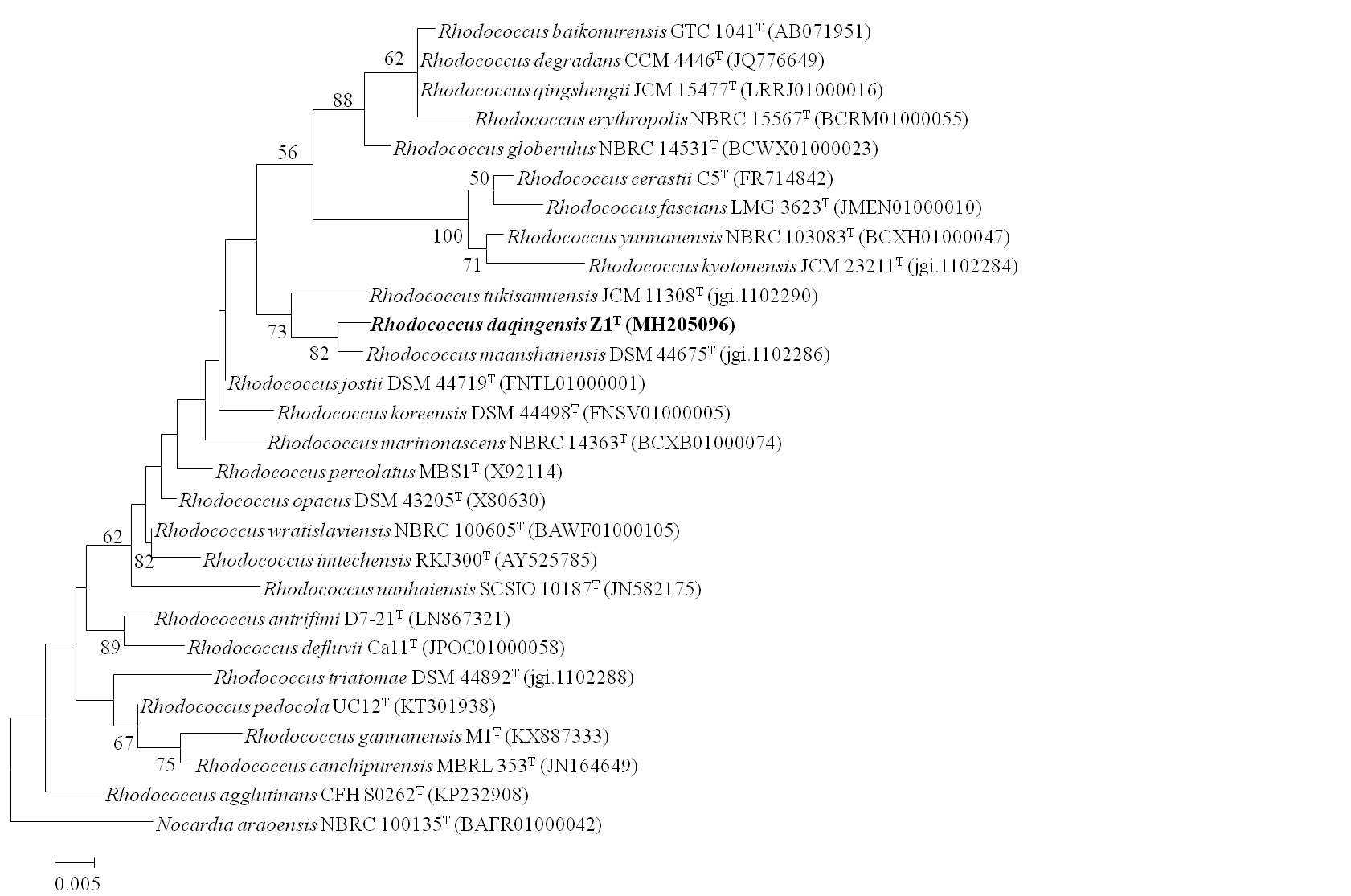
**

**a**


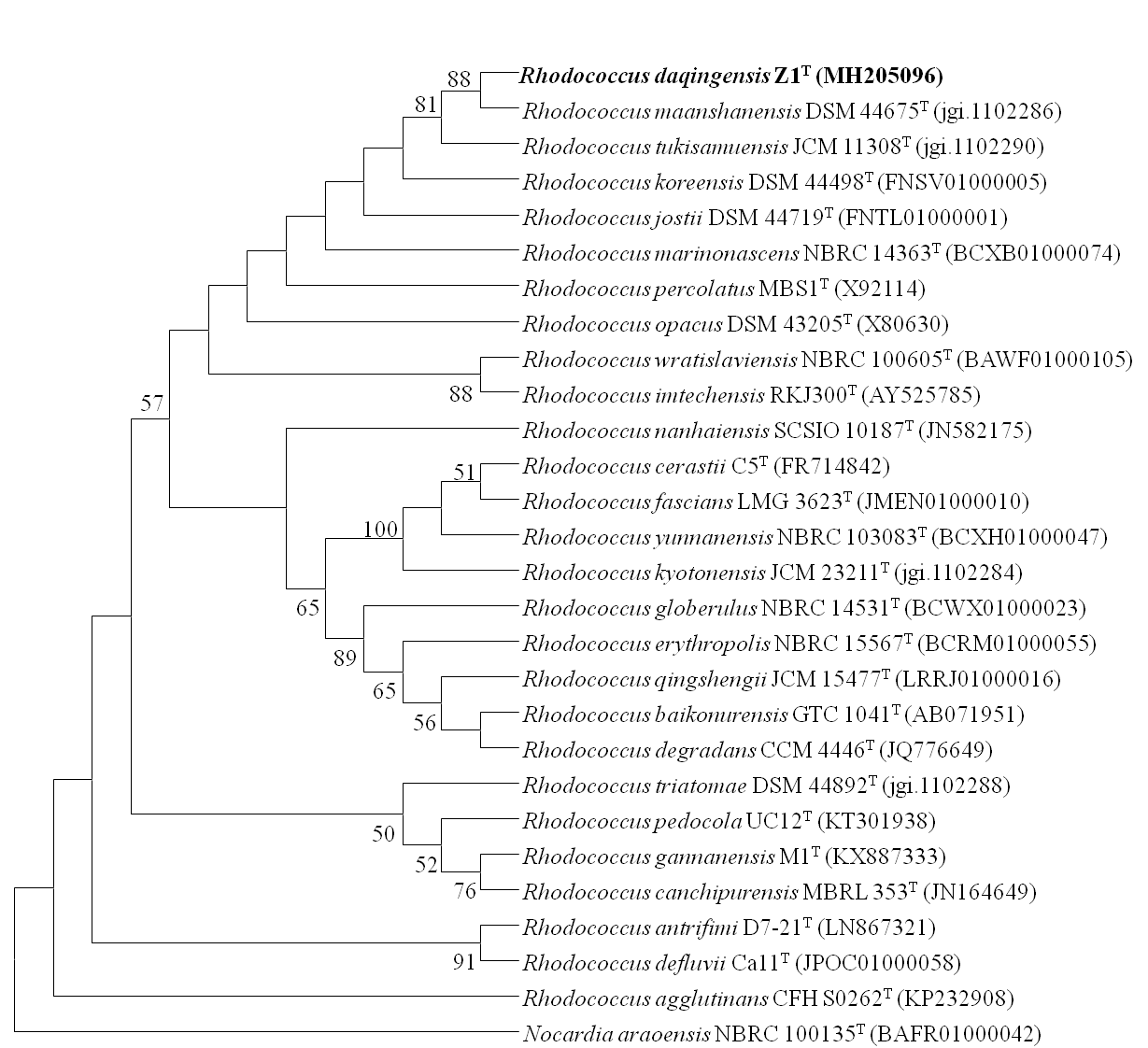
 **b**
